# Robust and Accurate Modeling Approaches for Migraine Per-Patient Prediction from Ambulatory Data

**DOI:** 10.3390/s150715419

**Published:** 2015-06-30

**Authors:** Josué Pagán, M. Irene De Orbe, Ana Gago, Mónica Sobrado, José L. Risco-Martín, J. Vivancos Mora, José M. Moya, José L. Ayala

**Affiliations:** 1 Computer Architecture and Automation Department, Complutense University of Madrid, Madrid 28040, Spain; E-Mails: jlrisco@ucm.es (J.L.R.-M.); jayala@fdi.ucm.es (J.L.A.); 2 Electronic Engineering Department, Technical University of Madrid, Madrid 28040, Spain; E-Mails: mirene@die.upm.es (M.I.D.O.); josem@die.upm.es (J.M.M.); 3 Neurology Service, Sanitary Research Institute, University Hospital La Princesa, Madrid 28006, Spain; E-Mails: dra.anagago@gmail.com (A.G.); sobrado.m@med.ucm.es (M.S.); jvivancos@neurogps.com.es (J.V.M.)

**Keywords:** migraine, WBSN, modeling, N4SID, prediction, robustness

## Abstract

Migraine is one of the most wide-spread neurological disorders, and its medical treatment represents a high percentage of the costs of health systems. In some patients, characteristic symptoms that precede the headache appear. However, they are nonspecific, and their prediction horizon is unknown and pretty variable; hence, these symptoms are almost useless for prediction, and they are not useful to advance the intake of drugs to be effective and neutralize the pain. To solve this problem, this paper sets up a realistic monitoring scenario where hemodynamic variables from real patients are monitored in ambulatory conditions with a wireless body sensor network (WBSN). The acquired data are used to evaluate the predictive capabilities and robustness against noise and failures in sensors of several modeling approaches. The obtained results encourage the development of per-patient models based on state-space models (N4SID) that are capable of providing average forecast windows of 47 min and a low rate of false positives.

## 1. Introduction

The continuous development of high-performance microprocessors and novel communication protocols has stimulated great interest in the research of wireless sensor nodes for wireless body sensor network (WBSN) applications [1]. They allow physiological signals to be easily monitored. In this study, wired sensors attached to the patient's body and connected to a wireless central node are used.

The most common WBSN application areas are healthcare in the elderly population, remote medical diagnosis, sport training mobile applications [2], disease alarm notifications, as Alemda *et al.* relate in [3] or rehabilitation [4]. The importance of promoting the use of these networks is shown in the amount of inversions made in projects like the “HealthService24 Project”, most of them in countries with elderly populations with chronic diseases [5]. One of the biggest areas of interest, because of its major impact on the user's satisfaction, is the use of data collected by WBSNs for prediction in chronic diseases. Some studies, for instance, have tackled the predictive functionality for epileptic seizures or anomalies in blood glucose levels. However, most of these studies have not dealt with the issues of real-time data acquisition by WBSNs, but from off-line processed databases.

One of the major problems with WBSNs is data loss due to disconnections, sensor problems or sensor losses and battery life; very common problems in ambulatory studies for ambient assisted living (AAL) [6]. Data losses pose strong demands on the real-time analysis of data, but the prediction capability should not be affected.

Migraine is a chronic recurrent headache, lasting several hours, and affects around 10% worldwide. Migraine is considered one of the most disabling diseases with a great socioeconomic impact. In this scenario, migraine patients would benefit from a crisis prediction application based on WBSNs, and pharmacological prophylaxis would reveal effective, increasing the quality of life and decreasing recurring costs. However, scientific studies so far have not established the hemodynamic changes that occur before a migraine attack. Moreover, as far as the authors are concerned, the work presented here is an innovative approach in several areas of pervasive health monitoring for migraine patients, and for the first time: (i) we perform continuous ambulatory monitoring of hemodynamic variables of migraineurs; (ii) this work makes a multivariable study of hemodynamic changes before, during and after a migraine attack; (iii) a study on the predictability of migraine attacks is presented.

In a real scenario of ambulatory monitoring of migraineurs, we pose and answer the following questions: Is it possible to predict a migraine? Can this prediction be launched in a specific time before the pain arrives? Does a migraine model exist? Is this model only per patient? How do these models respond under unreliable data acquisition? This paper proposes to find multivariable patient-based models for the prediction of migraine attacks. These models should be robust and accurate in an ambulatory scenario, where the reliability of data acquisition cannot be ensured. This real scenario brings data collected from a WBSN with all of the real problems of noisy data, data loss and sensor disconnections.

The remainder of this paper is as follows. Section 2 introduces the reader to the related work of the topic. Section 3 explains the methodology followed to gather the data and its management. Section 4 briefly describes the algorithms used and their parameters according to our problem. Section 5 shows the results obtained. Finally, some conclusions of this work are drawn in Section 6.

## 2. Related Work

Mobile health (m-Health) enables monitoring the type, quantity and quality of everyday activities improving daily care and the design of more clinically meaningful interventions to establish evidence-based practices [7]. Within m-Health, WBSNs are one of the most promising tools for unobtrusive healthcare monitoring. Wearable sensors placed on the body surface enable registering physiological and environmental human conditions, such as electroencephalogram [8], blood pressure [9] and accelerometry [10], among others.

The majority of health case studies with WBSNs in the literature are performed under a controlled setting [11–14]. On the contrary, the data collected for this paper were acquired from an ambulatory monitoring scenario, where real migraineurs wore the sensors almost 24 h per day [15]. Thus, this study captures the full complexity and multi-dimensionality of a real health scenario. Healthcare data volumes are massive, and as a consequence, the focus of health monitoring systems is progressively shifting from the mere sensor reading to the treatment and processing of the data [16] and the development of intelligent algorithms.

Three are the main challenges of the intelligent algorithms in health data mining systems [16]: prediction, anomaly detection and diagnosis. For instance, support vector machines (SVM) have been used in arrhythmia and seizure detection [17]; hidden Markov models (HMM) have been applied for detecting anomalies in blood glucose levels [18]; decision trees and Bayes nets have been used in stress studies [13]; and artificial neural networks (ANN) in diabetes works [19]. Using ANNs, Babušiak *et al.* [20] predict the ECG signal for arrhythmia detection. With the same purpose, Grandl *et al.* use these algorithms in WBSNs in [21]. Other work tries to predict epileptic seizures through EEG [22] using damaged data, but these have not been gathered from a WBSN.

This paper focuses on the algorithms of prediction and correlation among signals. Both kinds of algorithms are frequently applied in the literature to static datasets, either clinical or online databases of sensor data [23,24], where there is no data loss and signals are less noisy. Therefore, the first difficulty in this study is to deal with a noisy real scenario from which the gathered data come. Thus, the robustness will be a very important requirement of the developed data processing.

### 2.1. The Migraine Disease

#### 2.1.1. The Disease: Social and Economic Impact

Migraine is a primary chronic headache, with a significant hereditary component. It is characterized by recurrent headaches, unilateral or bilateral, throbbing, moderate to severe intensity and typically worsens with exercise. It may be accompanied by sensitivity to noise, light and/or odors. Sometimes, migraines are preceded or accompanied by transient neurological symptoms (visual, sensory or speech), and they are called migraines with aura. Migraine's current management and treatment is normally pharmacological and depends on the chronification level of the disease [25–29]. When the patients take their medication affects the efficacy of the treatment. This is usually too late to avoid the start of the headache, and the patient suffers the migraine disturbances equally.

Some economic studies [30] pointed out in 2001 that around 10% of the population was suffering from migraine. In 2012, the World Health Organization (WHO) maintained that rate in its Fact Sheet No. 277. In Europe, migraines mean a cost of €1222 per patient per year [31].

#### 2.1.2. Migraine Prediction

In migraines, the autonomic nervous system (ANS) regulates the heart and respiratory rate, sweating and vasomotor activity, among others. However, there are still many unknowns about how the dysautonomia is affected in a migraine patient and whether it is the cause or a consequence. In patients with aura (perceptual disturbance experienced by the patient before the pain) or prodromic symptoms (subjective and unspecific perceptual disturbance), the arrival of future pain can be mostly assured, but the prediction latency is unknown and pretty variable, as the symptoms can appear at any time from 48 to 6 h before the onset of the migraine. This prediction is almost useless for treatment purposes. As the current research shows a different dysautonomia in every patient, the study of this paper will search a per-patient-based modeling technique.

There are no studies of sweating and oximetry that show any change between the baseline and symptomatic periods. With respect to the body temperature, there are contradictions between patients in the literature [32,33]. These findings support our search for per-patient models. Moreover, none of the previous studies included a systematic multivariable analysis of continuously acquired ambulatory variables. Finally, no studies have been found about the possibility to predict a migraine crisis using hemodynamic variables. As previously mentioned, the ANS, in migraines, regulates hemodynamic variables. For this reason, this study presents a way to predict migraines through these variables. No studies have been found about the possibility to predict a migraine crisis; only Houle *et al.* in [34] have attempted to find some trigger (or absence thereof) to predict the cessation of a migraine attack to ultimately help with the prediction of the attack itself using only a dichotomized stress scale. Luciani *et al.* in [35] or Waelkens in [36] use prodromic symptoms, such as changes in character, in appetite or sleep rhythm as predictors of migraines. However, these predictors are non-specific and are subjective markers that can appear in patients' daily lives for other reasons.

There are also some studies about the early intake of medication to abort the crisis. Goadsby *et al.* in [37] demonstrate that the earlier the intake, the more effective. In addition, Hu et al. in [38] demonstrate that specific migraine treatments, such as rizatriptan, can abort the migraine in 30 min. Other specific treatments, such as sumatriptan, reduce this time to as little as 10 min before the crisis starts.

The study we present in this paper focuses on four hemodynamic signals: heart rate (HR), electrodermal activity (EDA), skin temperature (TEMP) and peripheral capillary oxygen saturation (SpO2). A multivariable analysis of these signals was used in an attempt to predict a migraine crisis.

Activity-related information is collected and used to distinguish between a migraine and other kinds of headaches. A robust patient model was developed in order to reduce the loss and the noise of data associated with ambulatory scenarios. According to Hu *et al.* in [38], this work is going to tackle the predictivity up to 30 min.

## 3. Experimental Scenario

One of the distinctive characteristics of the work presented in this paper is the experimental application of WBSN monitoring to one of the most wide-spread neurological disorders: the migraine. Unlike cardiovascular or movement disorders, this pathology poses several difficulties in the monitorization using WBSNs due to the hemodynamic changes that can occur before, during and after the migraine attack. In the literature, no previous studies have monitored hemodynamic variables continuously from the beginning to the end of a migraine, nor have they sought to predict migraines using multi-correlated variations in these data.

### 3.1. The Experimental Design

The hemodynamic variables are monitored during 24 h. To create prediction models, it is considered that, at least, more than 8 migraine events are needed. To achieve this, the patient is under study for approximately 4 to 6 weeks (this depends on the number of migraine attacks registered). The developed models will correlate changes in the input variables (the hemodynamic variables) to changes in an output variable, in this case, the symptomatic curve.

Once the patient signs the corresponding informed consent (the study protocol was approved by the Local Ethics Committee of the hospital), engineers and doctors of the group teach the patient how and where to place the sensors; they will not require a healthcare provider. The medical staff of the hospital selects the body locations where the sensors are placed. The position of the ECG sensors must be on the breast, near the heart. The SpO2 sensor is located on a finger. EDA can be measured in several locations; this sensor is located on the arm near the temperature sensor (near the armpit), to join the sensor's wires.

The patient wears the WBSN continuously during the day. If the patient has awakeness migraines or migraines when he sleeps, the monitoring process also takes place during the night. During the monitoring process, patients do not change their daily life. If it is required for some activity, such as certain sports, the monitoring process is stopped. The patients replace the sensors when the monitoring restarts. The patients also try to avoid any drug ingestion that can alter the monitored signals during the monitoring process. This is crucial in order to register the real body response before and during a migraine crisis. The patient will be allowed to take some medication if a strong migraine occurs. In these cases, the hemodynamic variables and the pain evolution are also collected; nevertheless, how the real pain would end without the effect of medication will be unknown, due to the effect that medications will accelerate the end of pain.

Each migraine episode has a different duration. For the modeling process, the data acquired during some hours before the patient marks the start of pain are also analyzed. This is necessary to identify changes occurring in the monitored variables before the attacks start. Approximately, 75% of the monitored migraine episodes are chosen in a random way for training and validation of the models. The remaining migraine episodes are used to test the models.

As previously mentioned, this study tries to find the prediction of the migraine attacks per every single patient. Therefore, from this point, the data and results presented belong to a single patient (Patient A). In Section 5, the whole study is repeated for other patient (Patient B) in order to compare the results and draw conclusions in Section 6. Data from Patient A correspond to a young female patient that suffers migraines with aura and is not under medical treatment. Twenty migraines have been acquired in two different experimental periods (almost a month each). The variables acquired in real time by our experimental setup comprise the four hemodynamic variables previously mentioned, and the subjective pain was collected from the patient by an electronic form.

### 3.2. Data Acquisition

**Figure 1:**
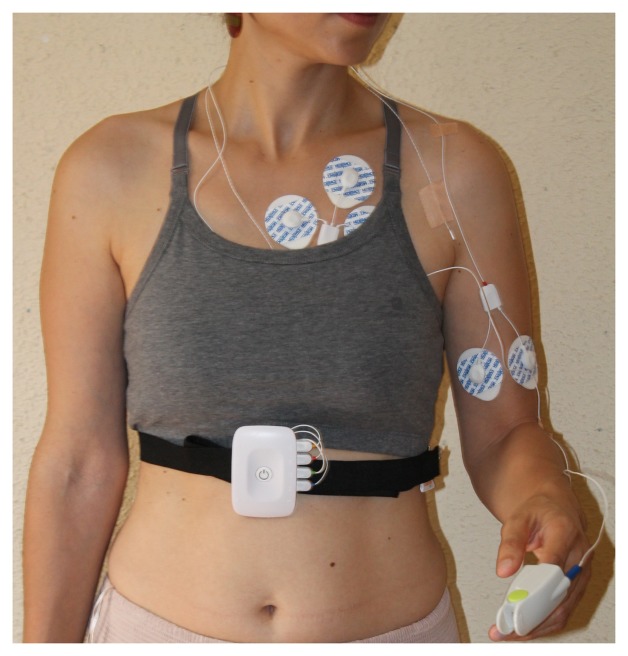
Patient wearing the monitoring kit.

The data acquisition was carried out with a WBSN integrated into a broader multitier telemedicine system. The architecture implemented involves two sensing motes, which communicate with an Android smartphone via Bluetooth. Data are stored and transmitted through the Internet to a cloud storage system. Follow-up and processing tasks are done on a remote PC or server. Figure 1 shows a patient wearing the monitoring kit.

The two used sensing motes are PLUX-Wireless Biosignals [39] and Nonin Onyx II [40]. EDA, skin temperature and ECG signals are acquired with the former and SpO2 values with the latter. The HR signal is calculated offline from the ECG signal by our peak-detection algorithm. The algorithms for migraine prediction will be developed from these four hemodynamic signals.

The Bluetooth-enabled Android device receives the data wirelessly as soon as each sensing mote takes a sample. The data acquisition sampling frequency for the PLUX-Wireless Biosignals sensors is 250 Hz for ECG and 1 Hz for TEMP and EDA, whereas the maximum sampling frequency allowed by the Onyx II device for the SpO2 is 3 Hz. This introduces some synchronization issues that need to be dealt with. All data have 12 bits of accuracy for the PLUX-Wireless Biosignals and 8 bits for the Onyx II. At these sampling rates, almost 1.5 MB of data are gathered per hour. A decimation of 1 min per sample has been established for data (TEMP, EDA, HR and SpO2) representation and usage.

The Android smartphone receives and temporarily stores the gathered data and also includes a form with questions related to every headache. These questions include the time when the migraine phase begins and ends, suffered symptoms and possible triggers of the attack. The patients are asked to fill in the form as soon as a headache has finished. They also are asked about the relative changes in pain intensity or punctual pain levels during the migraine crisis.

### 3.3. The Symptomatic Pain Curve

It is worth highlighting that the pain intensity depends on the patient; in this study, each patient evaluates his or her pain in two ways: a global index of pain for the total migraine period and punctual pain levels continuously marked during the migraine attack.

According to the global index of pain, there are multiple methods to evaluate the pain suffered by a patient, but all of them have the limitation of being subjective. We have chosen a limited numbered scale to represent the global pain, where 0 is no pain and 10 represents the highest (for each patient's scale). There are three categories for this scale: mild (1–3), moderate (4–6) or severe pain (7–10), in a normalized scale 0–10 [41].

According to the punctual pain levels along the migraine attack, we have chosen an unlimited numbered scale where 0 is no pain, and in the evolution, the marked points grow and fall according to the perception of the patient. If the pain increases, the patient marks a positive number, negative if it decreases and 0 if it remains equal. Thus, a curve of subjective symptom evolution can be drawn. The maximum represents the highest pain, and it will be different for each migraine. The unlimited range responds to the ignorance of the patient who does not know if the maximum pain has been reached or not. With a limited scale, there could be several saturated points to the maximum of the scale along a period of time. With our method, consequently, the curve of its evolution must be normalized in amplitude. The intensity varies along a migraine episode, usually starting with low levels, but depending on the patient, it can reach its maximum intensity in a variable way; the same occurs with its disappearance. Therefore, the pain curve must be individualized by each patient.

**Figure 2:**
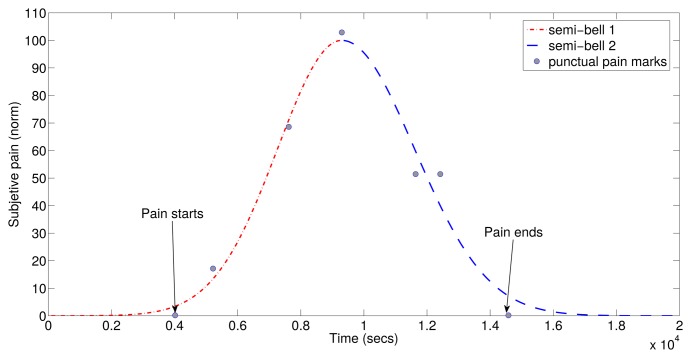
Modeling of subjective pain evolution curve.

To predict the symptomatic crisis, the first step is to generate a model of the migraine pain. To do this, an adjustment process of the registered subjective pain curve was carried out. The symptomatic curve has been modeled as two semi-Gaussian curves, as they fit the patient's subjective response. In addition to the punctual point of the pain evolution, patients also indicate three timestamps during the migraine attack. The first timestamp indicates the beginning of aura; the second one indicates the pain when detected; and the third one indicates the end of pain. With these three points and the punctual points of the pain evolution, two semi-Gaussian curves can be generated. {(*μ*_1_, *σ*_1_), (*μ*_2_, *σ*_2_)} are the two semi-Gaussian's parameters necessary to define a symptomatic curve. The symptomatic curve includes the aura and the pain period, as the latter reflects some changes in the migraine process. An example of the resulting function of is shown in Figure 2.

## 4. Modeling Techniques

The objective of this section is to explain the modeling system created to predict the symptomatic crisis of a migraine sufferer. Different modules compose the modeling system in the training and validation stage. This stage provides a set of models to work with. Following stages, such as the test stage and the final stage (a real time stage), will be presented in Section 5.

The N4SID algorithm is selected for this study. N4SID is a state-space-based algorithm. Modeling and predictive capabilities, as well as robustness against sensor problems have been studied in the N4SID models. The algorithm has been computed using the System Identification Toolbox of the MATLAB software [42].

In the following, the metric used to evaluate the accuracy of the models is the fit, defined as:

(1) $$\text{fit}=100\times \left(1-\frac{\left\|y-\hat{y}\right\|}{\left\|y-\text{mean}(y)\right\|}\right)$$

*y* is the real (Gaussian modeled) symptomatic curve, and *ŷ* is the predicted one.

### 4.1. Training and Validation Stage

**Figure 3:**
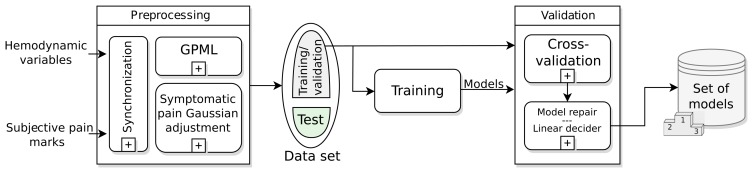
Training and validation diagram. This stage provides a set of models with which to work.

The training and validation stage, shown in Figure 3, is the first step to find the model or models that describe the symptomatic pain curve of a migraine sufferer better. This stage runs offline and has as inputs the data recorded in the monitoring: hemodynamic variables and subjective pain marks for every migraine attack. The most important modules in this stage are: the preprocessing, the training and validation dataset, the training and validation modules and the batch of models achieved.

#### 4.1.1. The Preprocessing Module

The WBSN can suffer failureof sensors or data can be lost due to Bluetooth disconnection or battery discharge. There are several ways to repair broken data. In this work, a synchronization subprocess sets a common time interval between samples using a high-order FIR filter decimator. The time between samples is 1 min for all signals and prediction horizons, unless something different is mentioned. After the synchronization, a Gaussian process machine learning (GPML) subprocess has been added in order to recover disruptions in data. This subprocess has been developed by Rasmussen [43], and the code is released under the FreeBSDLicense in [44]. In the case of the HR variable, this is calculated from the recovered ECG signal, and then, it is introduced again in the GPML subprocess. As mentioned in Section 3.3, a Gaussian symptomatic curve is modeled from the punctual pain levels marked along the migraine attack; this is the second subprocess in the preprocessing module. From this point, the modeling system is directly fed with the raw data TEMP, EDA, HR and SpO2.

#### 4.1.2. The Dataset

After preprocessing all of the gathered data, a set of data is available for model training. This dataset is randomly divided into two groups: around 75% of the monitored migraine episodes (*T*) are chosen for training and validation of the models, and the remaining 25% are used to test the models.

#### 4.1.3. The Training Module

As previously mentioned, the algorithm chosen for modeling and prediction is the state-space based N4SID algorithm. State-space models define immeasurable states to describe difference equations that calculate the current and future outputs from past and current inputs. N4SID is defined as a combined deterministic-stochastic model with the following parameters in Equation (2):

(2) $$\begin{array}{r} x_{k+1}=Ax_{k}+Bu_{k}+w_{k}\\ y_{k}=Cx_{k}+Du_{k}+v_{k} \end{array}$$

*u*_k_ are the *U* inputs and *y_k_* are the *Y* outputs at time *k*. *A* is the state transition matrix, *B* relates the states at time *k* (*x_k_*) with the inputs, *C* is the state to output matrix and *D* in the most of the cases equals zero. *v_k_* and *w_k_* are immeasurable white noises. In our case *U* = 4 inputs, *Y* = 1 output and the N4SID order *nx* (size of the square matrix *A*) has to be chosen as the best one in terms of fit.

As many of the model parameters have to be selected (past inputs, output and *nx* order), we compute several alternatives to select that with the best fit. This training stage is computed just once per patient.

Therefore, we define a time window of up to 100 min backward in steps of 5 min and a fixed horizon of 30 min forward according to the pharmacokinetics, as mentioned in Section 2.1.2. Thus, 20 different combinations of past-future horizons are performed. Furthermore, these 20 combinations are computed with *nx* from 1 to 10. We compute 200 combinations per migraine to find the best model. We select *T_i_* migraine events, *i* = 1, 2, …, *T*, for the training dataset. In this paper, we have used *T* = 15 Patient A's migraines for training and validation.

Finally, the current module provides a batch of (*M* = *T*) models (one per migraine attack) to the validation module. Not all of these *M* models are going to be used in the final prediction stage; just the best ones are selected. The work done shows that the selection of these best models in the training time is not the best solution for our work, as the traditional modeling studies do. The best models to make predictions are going to be selected in the validation module, as shown later in Section 5.

#### 4.1.4. The Validation Module

This module looks for the best models to predict migraines using the cross-validation criteria: each model *M_i_, i* = 1, 2, …, *M*, obtained from the *i*-th migraine is going to be validated against the other *j*-th migraines, with *i* ≠ *j*. The validations are made for different horizons, drawing a prediction curve. Each *M_i_* model performs *M* − 1 prediction curves. As the models were trained for a fixed prediction horizon of 30 min, the higher horizon, the worse fit.

The predictions obtained with the N4SID models have problems maintaining a constant value, and they tend to oscillate around the zero value when no symptomatic crisis are detected. This fluctuation causes an artificial reduction in the fit. Since these oscillations can be easily detected, we explain the solution adopted in the model repair submodule to solve this issue and improve the fit metric.

The model repair submodule must detect false positives and correct them. Hence, the aim of the model repair submodule is to detect and remove false positives in the predicted symptomatic curve during the validation. The false positive correction works as follows in an iterative process:

- A time window of the predicted curve is selected.
- A symptomatic threshold is applied. Predicted values above 50% of the probability of pain in the symptomatic curve are marked as positives using the linear decider co-subprocess described below. As these initial values are supposed to define a migraine attack, a forward search of points higher than this threshold is followed.
- After that, a time condition is applied. If the distance between the farthest points is lower than a supposed time window, then a false positive is detected and removed. This time window threshold has been set to 60 min, enough to detect if a migraine attack occurs or not.
- Once the migraine candidates have been found, the end and beginning of each event are chosen by detecting the left hand-side and right hand-side crossings of the symptomatic curve with the zero axes (Marks A and B, respectively).
- The time window is now shifted to start on a new timestamp in the future (Mark B), and the above steps are repeated sequentially.

At this point, the criteria for considering a pain intensity as migraine crisis must be defined; this decision is performed during the model repair subprocess. The linear decider subprocess is such a decision subprocess, which will detect a migraine event when the probability of occurrence of a migraine episode exceeds a threshold. This linear decider ranges from 0% (minimum pain intensity in the normalized symptomatic Gaussian curve) to 100% (maximum pain intensity in the normalized symptomatic Gaussian curve) of probability (see Section 3.3). Therefore, the linear decider projects the prediction made to a probability of occurrence of a migraine episode, with the linear function as the projection function.

This method improves the prediction horizon in several minutes for the best cases. In the worst case, the fit does not change. If this repair subprocess was not performed, all of the false positives would be included in the validation; the fitness may be low, and the higher prediction horizon, the faster the fit decreases. With this, some abrupt fitness breaks occur when the repaired model is not capable of detecting false positives. Nevertheless, this still remains at a higher fit than not using this model repair subprocess for the same prediction horizon.

At the end of this module, we obtain *M* models validated for different prediction horizons over *M* − 1 migraines.

#### 4.1.5. Set of Models

The aim of this selection is to make more robust predictions. It is supposed that just one model to predict all of the migraines can underestimate or overestimate the input data. With this idea, a set of models is going to be selected from the previously trained and validated batch. To do this, a ranking of models is provided: the models are sorted in relation to the average prediction horizon achieved in their validation. The best models are chosen. According to our experimental set, the selection of one third of the models (*M_best_* = *M*/3) is considered good.

Each *M_best_* model will generate a prediction; the prediction result in the test and real-time stages will be calculated as the average of all of the *M_best_* predictions performed (see Section 5.3).

## 5. Results and Discussion

In this section, the results obtained with the N4SID algorithm are presented. The results of the training module (see Figure 3) are shown firstly. Secondly, we presents the results of the validation module in order to analyze the prediction capabilities of the batch of models obtained during the training stage. Here, a selection of the models is performed based on the validation results (see Section 4.1.3). After the validation, a test phase is run to analyze the capability of the generalization of the models. During the test phase, the same method followed in the model repair subprocess is applied to detect false positives (see Section 4.1.4 and Figure 3). Robustness against sensor failures is also checked during this stage. Some other tests with other migraines and baseline states are checked to analyze the detection of false positives.

In the test stage, a sensor-dependent model selection system (*SDMS*^2^) criteria is proposed, giving a way to deal with sensor failuresor saturations. Finally, a real-time scheme is proposed. It will provide the prediction response expected for the whole modeling system.

### 5.1. Repairing the Data: The Gaussian Process Machine Learning Results

**Figure 4:**
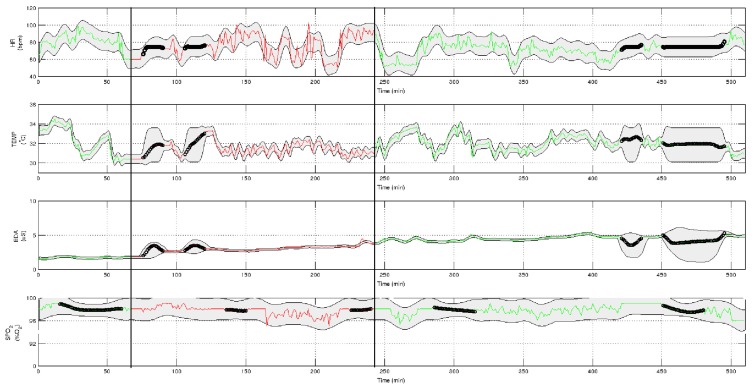
Gaussian process machine learning (GPML) and data synchronization applied during a migraine episode.

First of all, data from the four hemodynamic variables to work with are preprocessed in the preprocessing module (Figure 3). The result is shown in Figure 4. The green-colored signal sections are asymptomatic periods of time, while the red-colored sections represent the migraine itself (between black vertical bars). Some lost data have been recovered by the GPML subprocess. These data are represented with black points in the graphs. The grey zone represents the intervals of confidence of the GPML. From top to bottom, the signals have been recovered with the following fits: HR with 75.4%, TEMP 85.7%, EDA 93.2% and SpO2 73.4%. These fits are good enough; nevertheless, a finer tuning of the modeling parameters in the GPML could improve the results. This work will be tackled in the future.

### 5.2. Training: Creating the Models

**Figure 5:**
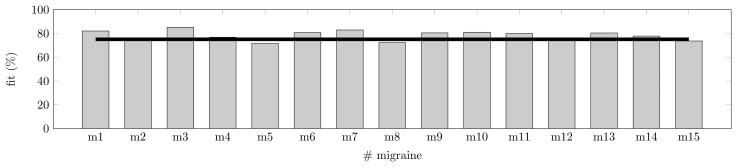
Fit for 15 randomly-chosen migraines and the average after training them with the N4SID algorithm and 30 min of a future horizon.

As shown in Section 3.3, a symptomatic curve can be modeled as the junction of two Gaussian curves. The training of the models has been performed for Patient A with *T* = 15 randomly chosen symptomatic crisis, as described in Section 4.1.2. The training has been run for the four predictors (features) available: TEMP, EDA, HR and SpO2. The training has been made for a fixed prediction horizon of 30 min; this time is justified by the pharmacokinetics in Section 2.1.2. In Figure 5, the fit metric shows how some migraines behave far better than others for the fixed horizon. Some models (six models) show a fit over or equal 80%, and none show a fit lower than 70%. On average, these models with four features are able to fit a migraine event with 75.1% accuracy.

Table 1 summarizes the parameters that perform the best fit for the search space proposed in Section 4.1.3. In Table 1, *f* is the fit achieved. *ph* and *nx* are the best past horizons (in {5, 10, …, 100}) and the best state transition matrix's order reached by the N4SID algorithm (in {1, 2, …, 10}), respectively. As previously mentioned, the future horizon is always 30 min. No correlation has been found between order, fit and past horizon; hence, some of the models achieve good fit values with low effort (low matrices' orders and short past horizon with high fit), such as model *M*_3_, while others require a higher effort, such as model *M*_8_; this with a high order, and the large past horizon reaches a 72.3% of fit.

### 5.3. Validation

Here, we validate each *M_i_* (*i* = 1, 2, …, 15) model obtained for each *j*-th migraine (*i* ≠ *j*). The validation is performed for different prediction horizons: from 1 to 100 min and keeping the best past horizon and best model's order calculated in Section 5.2. These validations have been obtained after repairing the models as explained in Section 4.1.4. The results are shown in Table 2 for fits at 70%. A fit of 70% has been considered a good threshold of similarity. The effort to reach fittings greater than 80% in the training stage is hard, as shown in Table 1, and values lower than 70% are considered not good enough; hence, this value has been selected as the reference or minimum fit value accepted and will be used from now on.

Models have been sorted according to the average horizon and then according to the minimum horizon required to ensure the higher minimums. Each model was trained with one migraine and 30 min of prediction horizon; hence, validation over other migraines will not reach this horizon, as they have not been used in the training of the model. The validation of one model generates 14 fitting curves with 100 points each, as previously mentioned. These curves reach 70% of fit at some point; these crosses lead to a minimum and a maximum horizon, and hence, an average value represented in Table 2.

**Figure 6:**
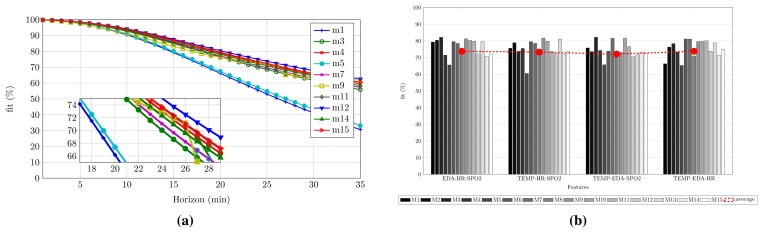
Validation applying the average model, and training for triads of features for Patient A. (**a**) Average model with *M_best_* models applied over the remaining 10 migraines; (**b**) Fitness comparison for N4SID and different three-features combinations in the training stage.

According to the results previously mentioned, we wonder if the best model, *M*_6_, is good enough for performing the migraine prediction. As seen in Table 2, the maximum horizon is not achieved by *M*_6_, but for *M*_11_. This possibility was taken into account in the design of the experimental setup (see Section 4.1), and a ranking of the models is performed. With this purpose, the best *M_best_* = *M*/3 = 5 models (first five models in Table 2), according to the selection made in Section 4.1.5, are chosen to define an average model. This works as follows: for each migraine, each model *M*_*best_i_*_ is applied. Five predicted symptomatic curve are achieved. The result is the average of these five validations for a given horizon. At the end, the false positives are removed with the model repair submodule. The result is shown in Figure 6a. Axis *x* represents the prediction horizon, meanwhile axis *y* represents the fit between the average prediction and the real symptomatic curve. In this process, the average, minimum and maximum horizons achieved at 70% are: 25, 18 and 28 min, respectively.

Notice that, while models *M*_1_, *M*_3_ and *M*_7_ exceeded 80% of the fit in the training stage, they have not been selected as part of the best models in the validation. This is due to these models being overfitted.

#### 5.3.1. Improving the Predictions

Is it possible to improve the prediction horizon? Does any feature worsen the fitness? To answer this, we follow a deeper analysis of the N4SID algorithm. In this case, we present the fit in Figure 6b for combinations of three features. As expected, triads have a lower fitness than the four features combination, and the fitness is even lower if duos are studied. However, as all of the three feature combinations still show acceptable fit values (close to the threshold of 70% explained in Section 5.3), they will proceed to the validation stage.

The same process has been followed for all combinations of three features—training, validation, ranking, best models selection—and, finally, the average model technique is applied. The results will show the most useful features to predict a symptomatic curve for Patient A. These results will also show the importance of the features in the prediction.

In Table 3, a summary of the results is shown. Now, we introduce data from Patient B in order to check how good or generalizable the training and validation stage developed is. Data from Patient B correspond to a middle aged female patient that suffers migraines without aura and without preventive medical treatment. Twelve migraines have been acquired in one experimental period (almost a month). The training dataset for Patient B was of eight migraine events; *M*_*best_B_*_ = 3 models have been chosen to create the average model.

The analysis of Table 3 brings meaningful results. First of all, we can observe how the inclusion of some features in the model worsens the prediction. For instance, for Patient A, the absence of the EDA features worsens the average prediction horizon in 2 min. On the other hand, the remaining three combinations of features maintain or improve the average and maximum prediction horizon. For Patient B, this does not occur in the same way. In this case, the worst scenario occurs for the four feature combination; in the others, the absence of one feature improves the prediction horizon. In addition, we can notice that the models of Patient B result in better predictions than the models of Patient A. The best result is achieved for the TEMP-EDA-SpO2 feature combination, with up to 52 min of a horizon and an average of 47 min. The remaining three feature combinations are almost as good as this. In most of the cases, the average horizons achieved exceed or they are close to the desired 30 min for both patients.

The analysis of these results shows how not only the selection of the acquired variables, but also that their combination in the matrices of the N4SID models can result in better or worse predictions. Furthermore, we can observe how the selection of features (the selection scheme) changes for every patient, providing a different set of models per patient; even when the best set of features is the same for both.

#### 5.3.2. Robustness: The Sensor-Dependent Model Selection System (*SDMS^2^*)

From the results obtained in Section 5.3.1, lets take the best feature combination, EDA-HR-SpO2 for Patient A and TEMP-EDA-SpO2 for Patient B, as well as their selected models *M*_*best_A_*_ and *M*_*best_B_*_. In this section, we force the models to extreme situations to evaluate the robustness against failing sensors (total or partial loss and saturation). Table 4 shows the average, upper and lower prediction horizons (in minutes) achieved in different situations of failing sensors (again, for a fit reference of 70%). As in Table 3, the *M* – *M_best_* migraine crisis (not used for the average model) is used to validate the robustness experiment.

As seen in Table 4, the best cases of prediction (34 and 52 min for Patient A and Patient B, respectively) are never reached in the case of lossy (variables equal zero) sensors (see Section 5.3.1) or saturation. Only for Patient B, the maximum is almost reached when the temperature and EDA sensors are saturated. The average horizons (25 and 47 min for Patient A and Patient B, respectively) are not exceeded either, and only for Patient B, the average horizon reaches 46 min when the EDA sensor is lost.

**Figure 7:**
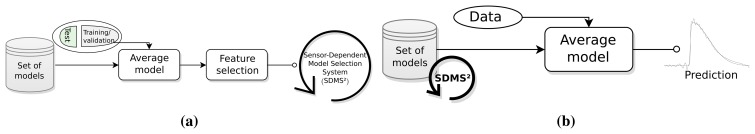
*SDMS*^2^ design and usage in the real-time application. (**a**) Sensor-dependent model selection system (*SDMS*^2^); (**b**) Implementation of the system for real-time applications.

**Figure 8:**
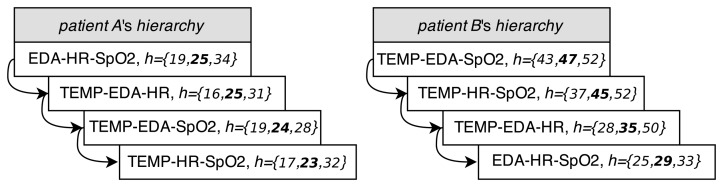
Hierarchies of sets of models for Patient A and Patient B.

At this point, we can introduce the sensor-dependent model selection system (*SDMS*^2^). This system, shown in Figure 7a, is able to detect saturated or lossy sensors. The *SDMS*^2^ senses the status of the sensors and chooses the best set of models according to their availability in real time. For each patient, at the validation stage, this system implements a hierarchy of sets of models, depending on the availability of sensors, after creating the whole set of models for every combination of features according to Table 3. The hierarchies of sets of models for Patient A and Patient B are shown in Figure 8. The ordination is represented from top to bottom; vector *h* represents the information of minimum, average (highlighted) and maximum horizons from Table 3.

Patients wear the four sensors along the day. The selected model (and set of features) for the individual will be applied to predict the migraines. If one of the sensors fails or saturates, then the next model according to the set of available features will be selected, trying to maintain the prediction horizon. It is possible to eliminate one of the sensors if the patient desires it; however, it is worth noting that, if one sensor fails or saturates, the prediction horizon will be lower, according to Table 4.

### 5.4. Test

In this section, we present some test results. All of the tests have been run with the *fh_average_* achieved for each feature combination (see Table 3) and applying the real-time stage in Figure 7b. The average model has been applied to the remaining migraine episodes—five for Patient A and four for Patient B—and several asymptomatic intervals. To evaluate the results the statistical *F_score_* is used. The *F_score_* is the harmonic mean of precision (or positive predictive value, PPV) and recall (or true positive rate, TPR), all of them defined as follows:

(3) $$TPR=\frac{T_{p}}{T_{p}+F_{n}}$$

(4) $$PPV=\frac{T_{p}}{T_{p}+F_{p}}$$

(5) $$F_{\text{score}}=2\frac{TPR\times PPV}{TPR+PPV}$$

**Figure 9:**
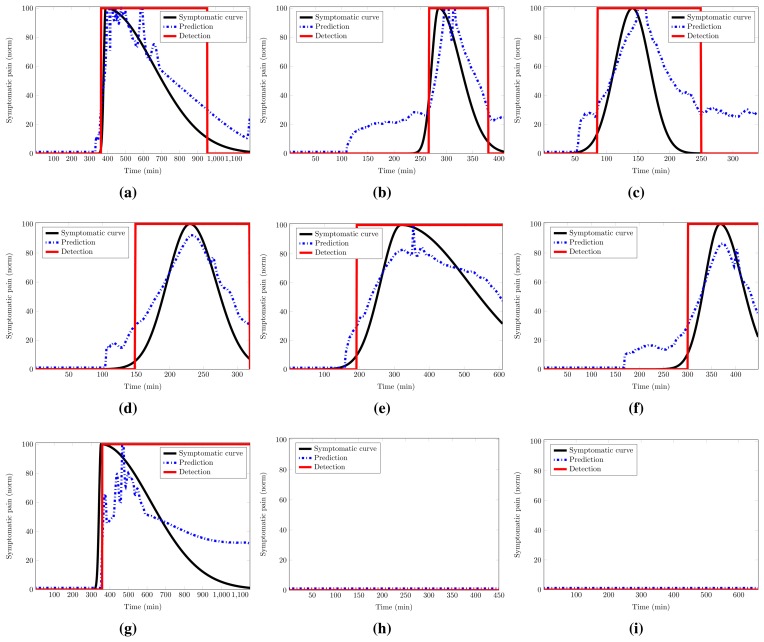
Test results for symptomatic and baselines periods for the trained patients. (**a**) Patient A, fit = 75.7%; (**b**) Patient A, fit = 55.0%; (**c**) Patient A → B, fit = 73.9% for TEMP-EDA-SpO2; (**d**) Patient B, fit = 88.9%; (**e**) Patient B, fit = 79.3%; (**f**) Patient B, fit = 81.1% for EDA-HR-SpO2; (**g**) Patient B → A, fit = 31.6%; (**h**) Basal Patient A; (**i**) Basal Patient A → B.

The TPR shows how many positive detections, *T_p_*, are found in the prediction against the false negatives, *F_n_* (those events not detected). The account of the detections is performed by the model repair submodule and the linear decider in Section 4.1.4. The PPV confirms how many of those detections are true. It is worth noting that the *F_score_* does not compare between the sets of features, but shows how good the selected set at the validation stage is. A true positive (*T_p_*) is considered when a detection is achieved and the fit in the migraine period is higher than or equal to 70%. This avoids spurious detections without a reliable fit (see Figure 9). As was described in Section 4.1.4, values above 50% of the probability of the pain curve are marked as positives. Spurious detections not removed by the model repair submodule are called false positives (*F_p_*).

The test has been run with the average horizons achieved in Table 3 up to a maximum of 30 min, the horizon expected to be reached according the pharmacokinetics, as mentioned in Section 2.1.2. Table 5 shows the results obtained for both patients. The confidence in the selected models is good enough for most of the sets of features. Only the TEMP-EDA-SpO2 combination for Patient A and the EDA-HR-SpO2 combination for Patient B present quite poor results. Avoiding these cases, in general, the TPR is above 70%. In addition, the PPV is 100% for both patients; so, the predicted migraines are all truly migraines. The best case for Patient A is for the TEMP-EDA-HR and EDA-HR-SpO2 combinations with an *F*_*score_A_*_ = 80% and for Patient B, the combinations TEMP-EDA-SpO2 and TEMP-HR-SpO2 with *F*_*score_B_*_ = 95%. This does not mean that these are the features that achieve the best horizons (as seen in Section 5.3), but they are the most reliable. Notice that the best set for both patients (EDA-HR-SpO2 for Patient A and TEMP-EDA-SpO2 for Patient B) are the most reliable ones. The less reliable ones are combinations of features allocated at the end (or nearly) of the hierarchy. None of the asymptomatic periods leads to false positives for any set of features.

#### 5.4.1. Generalization of Models for Other Patients

How generalizable are models for other individuals? Lets apply the models selected from one patient to all of the migraine events from another. Asymptomatic time periods are also taken into account, in order to check if baseline periods are equal for everyone. Again, a true positive (*T_p_*) is considered when a detection is achieved and the fit in the migraine period is higher than or equal to 70%. Results are shown in Table 6.

For Patient A and the combination of features TEMP-EDA-SpO2, models are capable of detecting some migraines from Patient B (a TPR of 44% means that this percentage of Patient B's tested migraines have been detected). Nevertheless, this does not occur again in other sets of features, and the remaining *F_score_* are quite low.

The results for Patient B are good enough: no detections with a fit higher than 70% lead to false positives during symptomatic periods. The maximum fit reached is 41.3% for the TEMP-EDA-SpO2 set of features. No asymptomatic period produces detections. For Patient B, if the calculated average horizons (larger than 30 min) are applied the TPR and *F_score_* fall to the 40% and 53% on average, respectively. This means that only around 40% of migraines will be detected. This low rate is accepted, due to these average horizons being quite high, and it is hard to generalize them.

These results support the initial idea that models must be trained per patient, as we assumed from the beginning in accordance with the fact that the behavior of the autonomous system depends on the patient. None of the asymptomatic periods led to false positives. This brings the conclusion that variations really appear in the hemodynamic variables measured before the pain starts, and these changes are patient dependent.

#### 5.4.2. Some Test Results

Figure 9 shows several results for the cases previously mentioned. All results correspond to the first set of features for both patients (EDA-HR-SpO2 for Patient A and TEMP-EDA-SpO2 for Patient B), unless something different is mentioned. Remember that all of the tests have been run with the *fh_average_* achieved in validation up to a maximum of 30 min. The blue dotted line represents the prediction response of the average model. The rectangular red solid line is the detection made by the linear decider. It provides the prediction at the 50% of probability of pain.

Figure 9a,b shows the best and the worst test cases for Patient A: 75.7% and 55.0% of fit, respectively. Notice that in Figure 9b, the migraine has been detected, but our metric, the fit value, evaluates this case as no detection, due to the delay in the detection. Figure 9c represents one of the detections achieved for the set of features (TEMP-EDA-SpO2) with the average model for Patient A over a migraine of Patient B. As can be seen, the prediction and the detection are good enough, and this leads to a high *F_score_* in Table 6, as explained in Section 5.4.1.

Figure 9d,c shows the best and the worst test cases for Patient B: 88.9% and 79.3% of fit, respectively. Figure 9f represents the best detection made for the set of features EDA-HR-SpO2 with models from Patient B over a migraine of the same patient. As previously mentioned in Section 5.4 and Table 5, this set is less reliable for Patient B; here is presented a good detection. Figure 9g represents one of the fault detections with the average model for Patient B over a migraine of Patient A. This detection has a fit of 31.6%; nevertheless, it seems that the prediction is good, at least for the start of the migraine; but the prediction is not able to reach the maximum and follow the decay, and it fails.

Figure 9h,i represents no detections over asymptomatic periods. In the first one, the average model from Patient A runs over an asymptomatic period of the same patient. The second one, on the other hand, runs over an asymptomatic period from Patient B.

As expected, the provided model seems be patient oriented and fails to predict the migraine for other patients who are not included in the training of the model for the metric (fit) used. However, this fact can be explained because the dysautonomia is differently affected in migraine patients, and a general model of the migraine cannot be found.

## 6. Conclusions

With a high accuracy, the modeling techniques developed in our research work are able to predict a migraine attack with a maximum horizon of up to 52 min (47 min on average) before it occurs at the validation stage. Additionally, an average prediction horizon of 30 min shows low variability, as well. Moreover, the experimental work has analyzed the robustness of the proposed techniques against failing sensors and has shown how a per-patient model has to be envisioned.

Migraine disease is one of the most common neurological disorders with a substantial cost per patient and a high rate of the reduction of productivity at work. Trying to find a more effective administering time for the pharmacological treatments that prevent the pain before it happens, this paper shows how a migraine can be modeled and predicted from some hemodynamic variables (features) gathered by a low-cost WBSN.

Pain has been modeled as a Gaussian curve from the subjective pain exhibited by the patients during their migraine attacks. A selection of features has been made, and a group of models has been selected in order to provide an average model. These models, obtained with the N4SID state-space algorithm, have been applied to four measured features: skin temperature, EDA, HR and SpO2, showing how the suffered pain can be characterized for the first time in the literature by these hemodynamic variables.

The achieved horizons of prediction can be considered useful and accurate times to anticipate migraine pain, more than the prodromic and aura symptoms that appear in some patients. Our current research work aims at increasing the horizons of prediction by developing advanced modeling approaches and applying this to a larger number of patients.

## Figures and Tables

**Table 1:** Best models' parameters found for migraines from Patient A with 30 min of future horizon.

	*M*_1_	*M*_2_	*M*_3_	*M*_4_	*M*_5_	*M*_6_	*M*_7_	*M*_8_	*M*_9_	*M*_10_	*M*_11_	*M*_12_	*M*_13_	*M*_14_	*M*_15_
*f*(%)	82.0	76.4	85.2	76.8	71.6	80.8	82.9	72.3	80.5	81.0	79.9	73.9	80.4	77.9	73.7
*ph* (min)	30	25	60	35	35	30	115	70	25	45	65	55	25	100	95
*nx*	8	8	6	8	7	10	9	9	10	8	8	10	9	5	7

**Table 2:** Validation for every *M_i_* model from Patient A. Future horizons for fit at 70%.

	M_6_	M_13_	M_2_	M_8_	M_10_	*M*_4_	*M*_15_	*M*_11_	*M*_9_	*M*_12_	*M*_3_	*M*_7_	*M*_5_	*M*_1_	*M*_14_
*fh_average_*	22	20	20	20	16	15	14	14	13	13	12	9	5	5	5
*fh_min_*	18	19	17	17	10	10	9	8	10	8	7	4	3	2	1
*fh_max_*	25	21	22	24	22	20	19	29	14	21	15	11	7	8	10

**Table 3:** Prediction horizons for all the combinations of features for Patients A and B. TEMP, skin temperature; EDA, electrodermal activity; HR, heart rate; Sp02, peripheral capillary oxygen saturation.

	**TEMP-EDA-HR-SpO2**		**TEMP-EDA-HR**		**TEMP-EDA-SpO2**		**TEMP-HR-SpO2**		**EDA-HR-SpO2**	

	**Patient A**	**Patient B**	**Patient A**	**Patient B**	**Patient A**	**Patient B**	**Patient A**	**Patient B**	**Patient A**	**Patient B**
*fh_average_* (min)	24	28	25	35	24	47	23	45	25	29
*fh_min_* (min)	19	17	16	28	19	43	17	37	19	25
*fh_max_* (min)	28	32	31	50	28	52	32	52	34	33
*fh_σ_* (min)	3	6	5	10	3	4	4	6	4	3

**Table 4:** Robustness analysis. Horizons of prediction for fit at 70%.

	**Patient A**						**Patient B**					

	**EDA (μs)**		**HR (bpm)**		**SpO2 (%)**		**TEMP (°C)**		**EDA (μs)**		**SpO2 (%)**	

	**0**	**25**	**0**	**100**	**0**	**80**	**0**	**35**	**0**	**25**	**0**	**80**
*fh_average_* (min)	22	23	11	18	11	20	11	38	46	41	11	28
*fh_min_* (min)	18	18	10	15	9	16	10	33	42	37	9	24
*fh_max_* (min)	26	33	11	19	12	30	12	45	50	46	13	30
*fh_σ_* (min)	3	4	1	2	1	4	1	6	3	3	2	3

**Table 5:** Trust levels on models selected for a fit reference of 70%. TPR, true positive rate; PPV, positive predictive value.

**Features**	**Patient A**			**Patient B**		

	**TPR (%)**	**PPV (%)**	***F****_score_***(%)**	**TPR (%)**	**PPV (%)**	***F****_score_***(%)**
TEMP-EDA-HR-SpO2	60	100	75	50	100	67
TEMP-EDA-HR	67	100	80	60	100	75
TEMP-EDA-SpO2	47	100	64	90	100	95
TEMP-HR-SpO2	53	100	70	90	100	95
EDA-HR-SpO2	67	100	80	40	100	57

**Table 6:** Generalization of models for other patients for the reference fit of 70%.

**Features**	**Patient A Models over Data from Patient B**			**Patient B Models over Data from Patient A**		

	**TPR (%)**	**PPV (%)**	***F_score_* (%)**	**TPR (%)**	**PPV (%)**	***F_score_* (%)**
TEMP-EDA-HR-SpO2	0	100	0	0	100	0
TEMP-EDA-HR	22	64	33	0	100	0
TEMP-EDA-SpO2	44	100	62	0	100	0
TEMP-HR-SpO2	33	100	50	0	100	0
EDA-HR-SpO2	33	73	46	0	100	0
